# Rejuvenation of bone volume with CGF and i-PRF in intra-osseous defects

**DOI:** 10.6026/973206300200085

**Published:** 2024-01-31

**Authors:** Koppu Sitamahalakshmi, Sai Krishna Bingi, Govardhani Krishna Kumar, Yanamala Bhuvanesh, Surapaneni Keerthi Sai, Subramanyam Parkavi

**Affiliations:** 1 Department of Periodontology & Implantology, Kamineni Institute of Dental Sciences, Narketpally, Nalgonda, India; 2 Department of Conservative Dentistry & Endodontics Kamineni Institute of Dental Sciences, Narketpally, Nalgonda, India; 3 Department of Periodontology & Implantology, Government dental college and hospital, Vijayawada, AP, India; 4 Department of Periodontology & Implantology, Government dental college and hospital, Vijayawada, AP, India; 5 Department of Oral Maxillo-facial Pathology, Kamineni Institute of Dental Sciences, Narketpally Nalgonda, India; 6 Department of Periodontology & Implantology, Government dental college and hospital, Vijayawada, AP, India

**Keywords:** Concentrated growth factor (CGF), injectable platelet rich fibrin (i-PRF), regeneration, bone volume, platelet concentrates generation

## Abstract

The risk of further periodontal breakdown increases with a deep intrabony defect. Non-surgical periodontal therapy could pose a challenge and surgical intervention is mainly required to manage the defect. Autologous platelet concentrates such as Injectable platelet rich fibrin (i-PRF) and concentrated growth factor (CGF) may improve surgical outcome due to its enrichment with growth factors. Total of 04 patients involved in this study. After conventional flap debridement of intrabony defects, CGF is placed in 2 patients and the other 2 patients received i-PRF in their respective intrabony defects. Volumetric analysis was done pre-operative and 6 months post operatively in both the groups. Bone volume is significantly increased in both CGF and i-PRF group but higher in CGF group when compared to i-PRF group has high regenerative and reconstructive growth factors which helps aids in early and high bone fill when compared to i-PRF.

## Background:

The primary consequence in humans is tooth loss, and its typical clinical feature is the degradation of bone and connective tissue in the periodontal region. [1] Teeth flaws cause poor mastication, which lowers the quality of life for people with periodontitis and hinders their ability to speak and look well, all of which are socially functional. [2] Sub gingival scaling and root planning (SRP) has been used as the primary technique to remove localized periodontal irritation, hence preventing the development of inflammation. [3] SRP cannot essentially address the ideal outcome of returning the periodontal tissues to their pre-existing state. [4] In order to create the ideal environment for periodontal tissue regeneration, guided tissue regeneration (GTR) in the 1980s involves creating a barrier membrane over intrabony defects to stop fibroblasts and epithelial cells from interlacing growth. [5] However, because of innate flaws beneath the barrier membrane; it is difficult to repair periodontal tissue entirely. [6] Periodontal flap surgery in conjunction with autologous, allograft, and xenograft bone has become a regular clinical procedure with the discovery of biomaterials and Blood clots can develop and expand inside these tissues. But bone induction's effectiveness is still uncertain. [7] Finding a useful strategy is essential to achieving the objective of treating periodontal intrabony defects as best as possible. Recent studies concentrating on the functional regeneration of non-renewable tissues based on autologous "regeneration agents" have increased attention to endogenous regeneration technology. [8] The use of autologous platelet concentrates (APCs) in endogenous regeneration technologies is highly recommended and APCs are widely used in all fields of oral therapy through the generation transition from platelet rich plasma (PRP) to platelet rich fibrin (PRF) due to their superior characteristics and ease of production. [9] PRF comes in a variety of forms, including i-PRF, Titanium PRF (T PRF), Advanced PRF (A PRF), and Standard PRF (SPRF). i-PRF is made from nine millilitres of the patient's own blood in test tubes, centrifuged at 700 rpm for three minutes, flow cytometry revealed that, of all the solid PRF-based matrices, i-PRF contains the greatest concentration of platelets and leukocytes. Additionally, a comparison between the total cell counts in i-PRF and other liquid blood concentrate systems like PRGF and PRP revealed that i-PRF had a much higher concentration of platelets, leukocytes, monocytes, and granulocytes than PRP. [10]

Using a specialized centrifuge, blood samples are centrifuged at varying, regulated rates to yield CGFs. It is possible to isolate a significantly larger and denser fibrin matrix that is richer in growth factors than is usually found in PRF or PRP by using different centrifugation speeds. A fibrin network composed of thick and thin fibrillar elements was recently seen, and numerous platelet cell elements were seen creating a cell aggregation that was caught within the fibrin network. [11] Their research revealed that TGF-β1 and VEGF are present in both the CGF and red blood cell (RBC) layers, indicating that a better CGF isolation process would maximize the quantity of growth factors in the CGF layer. Additionally, their findings revealed a significant proportion of CD34-positive cells in CGFs; CD34 has been shown to be crucial for angiogenesis, neovascularization, and vascular maintenance. [12] A report of sinus and alveolar ridge augmentation suggests that, in principle, CGFs have greater potential for tissue regeneration in clinical and biotechnological applications.[13] Nevertheless, this is not well supported by research. Therefore, it is of interest to assess the effectiveness of CGFs and i-PRF in the treatment of periodontal intrabony defects.

## Materials and Methods:

The current investigation involved four intrabony defects in four patients who met the inclusion and exclusion criteria listed below in order to be recruited for the study.

## Inclusion criteria:

[1] Patients in the 18-50-year age range.

[2] Individuals have a periodontal pocket that is at least 5 mm deep when probed.

[3] Individuals who exhibit intrabony defects on radiography that is at least 3 mm deep (the distance between the defect's base and alveolar crest).

[4] Individuals who do not have any underlying medical conditions preclude surgery.

[5] Patients six months before the initial assessment have not received any kind of regenerative periodontal therapy in the affected area.

## Exclusion criteria:

[1] Study participants were not allowed to have uncontrolled diabetes, anticoagulant medication, immunosuppressive medication, or other systemic disorders

[2] Expectant or nursing mothers

[3] Patients exhibiting inadequate oral hygiene before to surgery

[4] Smokers (above ten each day)

## Content:

Before the study started, all of the patients were informed about it and had to provide written, informed consent.

## Random assignments were made to place these patients in one of two groups:

CGF group: implantation of CGF plus open flap debridement group i-PRF group: i-PRF plus open flap debridement The current study included three visits over the course of six months: one before surgery, one during the procedure, and four after the procedure.

## Periodontal assessment:

Using a stent reference point that was fixed, the pocket depth was computed. To reduce distortion, all of the personalized acrylic stents were kept on the ready-made study casts for the duration of the study.

## Clinical gauges:

Using a UNC-15 probe on a synthetic occlusal stent, the following clinical measurements were taken and recorded to the closest millimetre. Fixed reference point to base of pocket (BOP) is known as FRP - BOP. Gingival margin (GM) is the Fixed Reference Point (FRP-GM). The Fixed Reference Point (FRP) is the apical boundary of the stent.

## Parameters related to clinical practice:

## Probing Depth:

The distance between the base of the pocket to the free gingival margin. It was computed by subtracting (FRP-GM) from (FRP-BOP).

## Indices:

Plaque index was recorded according to the criteria of Silness & Loe (Table 1). The PI was calculated by adding the total number of scores and dividing by the total number of surfaces present. Plaque index of 0.1 - 0.9 indicated good oral hygiene, 1.0 -1.9 indicated fair oral hygiene and 2.0 - 3.0 indicated poor oral hygiene. Gingival Index as described elsewhere [14] is given (Table 2). The tissue surrounding each tooth is divided into four scoring units, mesiobuccally, mid buccal, distobuccally and lingual. Each area was clinically examined, probed and scored based on Degree of gingivitis 0.1-1.0: Mild gingivitis, 1.1-2.0: Moderate gingivitis, 2.1-3.0: Severe gingivitis

## Radiographic parameters:

## CEJ to BOD:

This is measured from cement-enamel junction to base of the defect. Calculating linear bone growth and volumetric bone fill. The linear measurements CEJ to BOD were used to determine the linear bone growth Linear bone growth was calculated by subtracting CEJ to BOD at baseline from CEJ to BOD at 6 months. Volumetric bone fill was calculated three dimensionally by using CBCT In vivo software through volume rendering tool.

## Imaging techniques:

Before performing regenerative procedure, cone beam computed tomography imaging was taken to check the 3D architecture of the intrabony defect for better treatment planning and evaluate the measurements preoperatively at baseline and postoperatively after 9 months. CBCT Imaging Technique: All the CBCT (NEW CS 9000 System®) scans were taken by a single trained technician at baseline and 9 months. The voltage (90.00KV), Current (10.00mA), Exposure time (10.8 sec). The reference chosen to standardize the axial and sagittal planes was the bi-spinal line, coinciding with the vertical and horizontal planes, respectively. The reference employed to standardize the coronal plane was the line between infra-orbital points, named the infra-orbital line thus concluding the positioning of images over the three spatial planes. The sagittal and coronal sections were reconstructed after 6 months at the same axial slicing to that of the baseline.

## Treatment protocol:

## Preoperative protocol:

Eligibility for study participation was screened after the initial evaluation. The study procedure was thoroughly explained to the qualified patients, and those who agreed to take part in the research were enrolled after providing their informed consent. In order to create occlusal stents for the treated teeth and to generate study casts, the clinical history was documented and impressions were taken. Under local anaesthetic, hand curettes and ultrasonic equipment were used for scaling and root planing. If occlusion-related injuries were identified, occlusal correction was carried out. Four weeks later, a periodontal re-evaluation was conducted to validate the sites' appropriateness for this periodontal surgery investigation. Prior to surgery, patients who are a good fit for the operation are recommended to get a baseline CBCT.

## Procedure:

Initial Visit (0 weeks, baseline): In order to assess periodontal health, RAL, PD, PI, and GI were recorded. Only patients who maintained optimal oral hygiene (PI < 1) were eligible for surgery after their oral hygiene maintenance was assessed. The patient was instructed to swab the peri-oral tissues with a 5% w/v Povidone iodine solution and rinse the oral cavity with 10ml of 0.2% chlorhexidine digluconate solution. Using a local anaesthetic approach with 2% lignocaine and 1:1,000,000 dilutions of adrenaline, the operative area was rendered unconscious. In order to maintain a clean surgical site, the procedure was performed with the appropriate aseptic precautions and continuous aspiration. Mucoperiosteal flaps were lifted and incisions made in the buccal and lingual sulcular regions. The greatest amount of interproximal soft tissue was preserved with extreme caution. Using ultrasonic equipment and hand curettes, the flaws were completely debrided, and root smoothness was ensured through scaling and root planing. Both groups underwent open flap debridement; one group received CGF placements, while the other group received i-PRF placements. On the first day following surgery, patients were provided 500 mg of Amoxicillin eight hours a day for five days and 50 mg of Diclofenac sodium, an analgesic, eight hours a day. The subjects were instructed to take an analgesic in case they felt pain later. Appropriate post-operative instructions were given to the patients who included avoidance of brushing, flossing and chewing in the surgical site for 2 weeks. Entire treatment flowchart is given (Table 3).

## Results:

Both the groups show excellent difference and improvement at 6 months when compare to baseline in PI, GI, PPD. However, in inter group comparison between CGF group and i-PRF group, CGF group shows higher reduction in PI, GI, PPD from base line to 6 months (Table 4). Both the groups increase in bone volume from base line to 6 months. CGF group shows more bone volume from baseline to 6 months when compared to i-PRF group (Table 5).

## Discussion:

The current study evaluates the effectiveness of platelet concentrate generation, or i-PRF, and CGF in periodontal intrabony or osseous abnormalities. Because platelet concentrates are a rich source of growth factors, they can prevent bleeding, hinder tissue adhesion, encourage healing, lessen pain, and speed up the creation of new tissues. A biological product called platelet concentrate is made from the patient's own blood and offers advantages like less bleeding, less scarring, and serous fluid collection [15]. There is a very small body of research on the application of CGF in intrabony defect regeneration that compares it to i-PRF. Therefore, the efficacy of CGF and i-PRF in treating periodontal intrabony osseous defect was assessed in this study. For more precise radiographic measurements, CBCT volumetric study of intrabony flaws was performed.

In this investigation, PI, GI, and PPD were used to examine soft tissue. Six months after the baseline, parameters were measured. Reductions in PI, GI, and PPD levels from baseline to six months in both groups demonstrate progress in this regard. On the other hand, the CGF group has improved more than the i-PRF group. This could be the result of numerous growth factors that aid in the formation of new tissue, such as vascular endothelial growth factor (VEGF), platelet-derived growth factor (PDGF), and transforming growth factor-β (TGF-β) [16]. These growth factors adhere to a dense network of fibrinous scaffolds, which prevents early proteolysis of the growth factors and slows down their release. This ensures the best possible results for both short- and long-term wound healing.

Software was used to perform bone volumetric analysis using CBCT. The findings demonstrated an increase in bone volume from baseline to six months in both groups. When comparing the CGF group to the i-PRF group, there is a noticeable increase in bone volume six months from the baseline. This could be because CGF accelerates Osseo integration by increasing osteoblast growth and bone healing. CGF, which includes fibrinogen, growth factors, leukocytes, coagulation factors, endothelial growth factors, and platelets, facilitates angiogenesis and tissue remodelling. Because of its many benefits, which include promoting osteogenesis and wound healing, accelerating epithelial, endothelial, and epidermal regeneration, and having homeostatic and tissue healing qualities, CGF also lessens scarring. Its high leukocyte concentration confers strong antibacterial qualities, and it acts as a scaffold to promote cytokines and cellular motility. Because of its mouldable nature and robust interconnected fibrin network, it may be effectively suited to various shaped bony deformities. It speeds up bone repair and eliminates the need for titanium meshes or bone tack by trapping platelets and leukocytes in the fibrin network to release the growth factor. Because of its strong fibrin interaction, it reduces the formation of soft tissue and doesn't require any biochemical additions to prepare. Growth factors and bone cells-both essential for bone formation-are found in the mineral scaffold. These components stimulate cells. The above-mentioned results are in accordance with Yousef [17], Mohd Noh [18], Yao M [19].

## Conclusion:

One course of treatment that can be recommended to patients for the management of intrabony defect is regenerative therapy. While resorbable GTR remains the gold standard for regeneration, platelet concentrates like as i-PRF and CGF can be applied as an adjuvant to replace collagen membrane in periodontal regeneration without sacrificing clinical results. These findings may indicate that, in certain situations, a single application of CGF could have the same therapeutic value as a combination of grafting materials. This would support the use of CGF alone in the future, thereby avoiding the need for grafting materials. This is efficient in achieving a decrease in PPD and radiographic results such as defect depth in comparison to CGF alone. Therefore, it is preferable to CGF when treating intrabony osseous abnormalities. Additionally, it was found that CBCT was a superior substitute for invasive histologic assessment.

## Ethics approval and consent participate:

The Ethics Committee of the Government Dental College and Hospital

## Consent for publication:

Approved

## Funding:

There are none

## Figures and Tables

**Table 1:** Plaque index

**SCORE 0**	**No plaque in the gingival area.**
SCORE 1	A film of plaque adhering to free gingival margin and adjacent area of the tooth. The plaque may be recognized only by running a probe across the tooth surface.
SCORE 2	Moderate accumulations of soft deposits within the gingival pocket and on the gingival margin and / or the adjacent tooth surface that can be seen by the naked eye
SCORE 3	Abundance of soft matter within the gingival pocket and/or on the gingival margin and adjacent tooth surface.

**Table 2:** Gingival Index

**VISITS**	**TIME PERIOD**	**TREATMENT DONE**
I	BASELINE	GI, PI, surgical procedure , volumetric analysis , procedure, oral hygiene instructions
II	2 WEEKS	Suture removal, recording of any adverse events, oral hygiene instructions
III	6 MONTHS	GI, PI, surgical procedure, volumetric analysis , procedure, oral hygiene instructions

**Table 3:** Entire treatment flow chart

**Mild gingival inflammation, slight changes in color, slight edema, no bleeding on probing**	**Mild gingivitis**
Moderate inflammation, redness, edema, glazing; bleeding on probing	Moderate gingivitis
Sever inflammation, marked redness and edema, ulceration, tendency to spontaneous bleeding	Severe gingivits

**Table 4:** Plaque index, gingival index and periodontal pocket depth values from baseline to 6 months in both the groups

**Parameter**	**Group**	**Time**	**No of patients**	**Mean**
	i-PRF group	Baseline	2	1.9
		6 months	2	0.9
Plaque index(PI)	CGF group	Baseline	2	1.9
		6 months	2	0.9
	i-PRF group	Baseline	2	2
		6 months	2	1
Gingival index(GI)	CGF group	Baseline	2	2
		6 months	2	1
	i-PRF group	Baseline	2	8.0mm
		6 months	2	6.0mm
Periodontal pocket depth (PPD)	CGF group	Baseline	2	8.0mm
		6 months	2	4.0mm

**Table 5:** bone volume from base line to 6 months in both the groups

**Parameter**	**Group**	**Time**	**Mean**
Bone volume in cubic centimeters (cc)	i-PRF group	Baseline	1326.93
		6 Months	1399.32
	CGF group	Baseline	1618.73
		6 Months	
			1807.61
